# A Brief Evaluation of Antioxidants, Antistatics, and Plasticizers Additives from Natural Sources for Polymers Formulation

**DOI:** 10.3390/polym15010006

**Published:** 2022-12-20

**Authors:** Suzete Almeida, Sila Ozkan, Diogo Gonçalves, Ivo Paulo, Carla S. G. P. Queirós, Olga Ferreira, João Bordado, Rui Galhano dos Santos

**Affiliations:** 1 CERENA-Centre for Natural Resources and the Environment, Instituto Superior Técnico, Av. Rovisco Pais, 5, 1049-001 Lisboa, Portugal; 2 CQE, Institute of Molecular Sciences, Departamento de Química e Bioquímica, Faculdade de Ciências, Universidade de Lisboa, Campo Grande, 1749-016 Lisboa, Portugal

**Keywords:** bio-additives, antioxidants, antistatic agents, plasticizers, sustainable polymers

## Abstract

The circular economy plays an important role in the preparation and recycling of polymers. Research groups in different fields, such as materials science, pharmaceutical and engineering, have focused on building sustainable polymers to minimize the release of toxic products. Recent studies focused on the circular economy have suggested developing new polymeric materials based on renewable and sustainable sources, such as using biomass waste to obtain raw materials to prepare new functional bio-additives. This review presents some of the main characteristics of common polymer additives, such as antioxidants, antistatic agents and plasticizers, and recent research in developing bio-alternatives. Examples of these alternatives include the use of polysaccharides from agro-industrial waste streams that can be used as antioxidants, and chitosan which can be used as an antistatic agent.

## 1. Introduction

Polymers are among the most versatile materials due to their excellent cost-to-performance ratio, durability and adaptability. However, industrial and commercial exploration has recently raised several environmental concerns, gaining widespread attention. Some of the issues found during the industrial exploitation of typical polymers are the feedstock used, mainly derivatives from petrochemical sources and therefore with a larger ecological footprint. Another concern is the disposal of manufactured products, such as plastics, with some remaining in the environment for hundreds of years. Their disposal in waste management facilities has been reported to release, often to the atmosphere, toxic and controlled gases such as CO_x_ and NO_x_ [1]. In Europe, packaging accounted for 40.5% of all plastic usage in 2020, followed by building and construction (20.4%) and the automotive industry (8.8%) [2].

During manufacturing, additives can be added to plastics to enhance specific properties, including degradability resistance or accelerant, physical aspects, and mechanical strength. Some additives are antioxidants, antistatic agents, antifogging agents, emulsifiers, fillers, impact modifiers, lubricants, plasticizers, release agents, solvents, stabilizers, thickeners, and UV absorbers. Additives may be either organic (e.g., alkyl phenols, hydroxy-benzophenones), inorganic (e.g., oxides, salts, fillers) or organometallic (e.g., metallo-carboxylates, Ni complexes, Zn accelerators).

Initiators, inhibitors, lubricants, stereo modifiers, stabilizers, catalysts, co-catalysts or catalyst carriers are usually used in the polymerization stage. Typically, additives that bestow unique technical properties are applied at the finishing stage. Examples of such additives include slip agents, antioxidants, lubricants, mold releases, antistatics and plasticizers. Performance-critical additives such as foaming agents, antioxidants, impact modifiers, lubricants and flame retardants are applied at the compounding stage. The most crucial aspect is obtaining a homogeneous mixture of polymer and additive [3].

In 2021, the plastic additives market was valued at USD 45.6 billion, with a predicted annual growth rate of 5.6%. The market’s growth perspectives are mainly driven by the rapid development of emerging economies such as India and Brazil and changes in consumer habits due to increased urbanization [4].

There has been increased interest in bio-based additives research to reduce the polymer industry’s dependency on petrochemical feedstocks [5]. However, some considerations, such as the nature of their large-scale manufacture whilst maintaining reproducible and expected properties, must be considered to encourage the industry-wide deployment of bio-based additives. The cost of the finished product is another crucial element to be considered, since it must be competitive with the current petrochemical-based additives, otherwise creating an insurmountable barrier to their entry into the market [6]. Using bio-based additives expands the use of bio-sourced materials in the polymers industry, decreasing their carbon footprint by reducing their reliance on petrochemical sources while simultaneously improving their quality and increasing their useful lifecycle. A few examples of renewable bio-sources for additives are chitosan [7], lignin [8], polysaccharides [9] and carotenes [10].

The concept of a natural additive should be established at this point for the benefit of clarification. In line with those derived from fossil sources, natural additives are chemical compounds that can be extracted from plants, animals or minerals and added to polymers to enhance their properties. On the other hand, synthetic additives are obtained through a chemical or enzymatic reaction.

This assay will give a short and concise evaluation of some of the advances in selecting sustainable additives for polymers formulation, focusing on antioxidants, antistatics and plasticizers.

## 2. Antioxidants (AOs)

AOs are substances that inhibit oxidation or hinder reactions promoted by oxygen or peroxides as preservatives in numerous products. AOs are usually small molecules with sites that can react with free radicals and hydroperoxides produced during oxidation reactions. AOs in the chemical industry typically indicate compounds that delay the autoxidation of chemical products such as rubber and plastic. Radical chain reactions between oxygen and substrates mainly cause autoxidation. Effective AOs, such as sterically retarded phenols and amines, are radical scavengers that hamper the radical chain reactions [11] AOs are chemical compounds and their principal advantage is protecting polymers and plastics against thermal and photo-oxidative processes occurring during their natural ageing [12,13].

**Figure 1:**
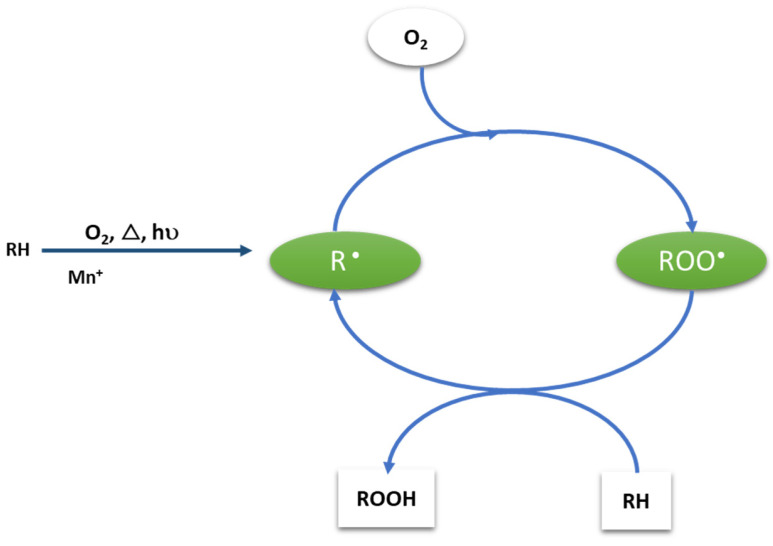
Mechanism of autoxidation cycle, adapted from Zweifel (1998) [14].

Zweifel (1998) proposed the autoxidation mechanism using the proper AO stabilizer. In Figure 1, the autoxidation cycle is schemed, highlighting the reactive species susceptible to the intervention of such additives in different reaction steps [14].

All AO species’ impact depends on factors such as chemical structure, the number of reactive groups, physical properties, AO concentration and environmental factors [15].

Many synthetic antioxidants are commercially available, although they are not very eco-friendly. Hence, great importance has been attached to identifying natural products with antioxidant activity. Some known natural antioxidants are chitosan, chitin and alginates and their derivatives [15].

Antioxidant activity of various natural products has been reported both in commercial polymers such as polyethylene (PE) [12,16] and polypropylene (PP) [17], and in bio-based polymers such as polylactic acid (PLA) [18,19]. According to Marturano et al. (2017), the studies prove that substituting petroleum-based products with their natural and renewable counterparts is possible in the future [12].

AOs encompass different classes of compounds that can intervene with oxidative cycles to inhibit or delay the oxidative degradation of polymers. Many additive classes have been developed to prevent or reduce the oxidative degradation of PP. These additives appear to work by a variety of mechanisms, some of which are hydroperoxide degradation and radical scavenging, to increase practical importance [13].

Total AO capacity depends on reaction conditions such as temperature, pressure, reaction medium, reference points and chemical reactivity. AOs are generally classed into two groups according to their protection mechanisms [13]. Classifications of some commercial AOs and their chemical composition are shown in Table 1.

### 2.1. Primary AOs (Kinetic Chain Breaking AOs)

Primary AOs are able to scavenge some or all of the free radicals and polymeric radicals present by donating a hydrogen atom through a process entitled the chain-breaking electron donor mechanism. Two classes of primary AOs can be broadly identified as radical scavengers (chain-breaking acceptors) and H-donors (chain-branching donors).

Chain-breaking AOs with reactive OH or NH groups (hindered phenols and secondary aromatic amines) inhibit oxidation through chain termination reactions. Inhibition occurs through the transfer of a proton to free radical species. The resulting radical is stable and cannot remove a proton from the polymer chain [13].

Secondary aromatic amines are the most efficient hydrogen donors and behave like primary AOs. Aromatic amines are generally more active than hindered phenols due to less steric hindrance and are available in various forms. In addition, aromatic amines change color more than hindered phenols when exposed to light or combustion gases [12].

### 2.2. Secondary AOs (Peroxide Decomposers)

Secondary AOs, typically sulfur- or phosphorous-based compounds, decompose peroxides that are intermediate products in the oxidation reactions and can regenerate the primary AO and split hydro-peroxy groups forming inert secondary products present in the polymer [12].

Secondary AOs, often called hydroperoxide decomposers, behave by converting hydroperoxides into non-radical, non-reactive and thermally stable products. They are often used with primary antioxidants to provide synergistic stabilizing effects. Hydroperoxide scavengers prevent hydroperoxides from decomposing into extremely reactive alkoxy and hydroxy radicals.

Phosphites, especially organophosphorus compounds, which are extremely effective stabilizers during processing and are typically used with a primary antioxidant, are secondary antioxidants that decompose peroxides and hydroperoxides into stable, non-radical products. Trivalent phosphorus compounds are excellent hydroperoxide decomposers. Generally, phosphites (or phosphonites) are used and react to form phosphates.

Among all the sulfur-based hydroperoxide disintegrants, thioethers and esters of 3,3-thiodipropionic acid are very important. The thio-synergist molecule is converted to various oxidized sulfur products, while hydroperoxide is typically reduced to an alcohol. Unlike organophosphorus compounds, thio-synergists are very efficient for long-term thermal ageing applications [13].

### 2.3. Natural AOs

Natural AOs are substances that prevent reactions such as spoilage, sourness and color change. Those are generally derived from plant sources and their activities vary depending on plant species, variety, extraction and/or processing methods, and growing conditions. Especially red-, orange- and purple-colored fruits and vegetables have high antioxidant activity. Tocopherols and tocotrienols, ascorbic acid, flavonoids, carotenoids and phenolic acids can be considered the most important natural AO groups [20]. Table 2 shows some of the AO components used for application in some polymers obtained from plant sources.

Natural AOs are used in many industries, including food, pharmaceuticals, fuels, lubricants and petrochemicals. In the food industry, natural AOs are widely used, e.g., β-carotene is used for restoring colours as a natural dye [22], and pectin, which is extracted from citrus peels, is used in the production of jams, jellies and thickeners, emulsifiers and stabilizers in dairy products [20].

Resveratrol is a naturally occurring AO, well known as a dietary supplement due to its potent antioxidant properties linked to anti-inflammatory activity. Agustin-Salazar et al. (2014) reported the effect of resveratrol on the thermal, thermos-oxidative and photo-oxidative degradation of PLA under the scope of the development of bio-based polymer formulations. Results showed that resveratrol catalyzes high-temperature transesterification reactions under an inert atmosphere but inhibits exothermal oxidative reactions of the polymer backbone [18]. Since resveratrol has little effect on the direct photolytic cleavage of ester bonds, it can slow the oxidative chain reaction of PLA through a peroxyl radical scavenging mechanism and is recommended as an environmentally friendly additive for a sustainable approach to the stabilization of PLA films in packaging and other applications [23].

Besides the food industry, natural AOs are also highly important for biodiesels. Biodiesel faces oxidative degradation over time due to the double-bonded fatty acids in its structure. The oxidative stability of biodiesel is affected by other factors, such as light, high temperatures, humidity, pigments, enzymes and metallic elements, during storage and commercialization. Therefore, using AOs, the biodiesel can be protected from autoxidation, thereby minimizing the formation of degradation compounds (for example, peroxides, aldehydes, ketones, dimers and polymers). AOs (phenolic compounds such as butylated hydroxy-anisole (BHA), butylated hydroxytoluene (BHT), propyl gallate (PG), and tertiary butylhydroquinone (TBH)) are used to prevent oxidation in biodiesel and other fuels. Natural antioxidants can improve oxidation stability and increase the biodegradable, non-toxic component of the fuel [22].

AOs such as vitamin E (tocopherols and tocotrienols) are primarily found in unrefined vegetable oils, but their concentrations and efficacy are often impaired after refining. The deliberate addition of natural AOs such as α-tocopherol or β-carotene to biodiesel has delivered good results [15].

The focus on tocopherols, vitamin C, carotenoids and phenolic compounds has led to the search for safe and effective naturally occurring antioxidants [24,26]. For example, tocopherols are components of Vitamin E that plants only synthesize and are found in seed oil, leaves and other green parts [16].

The application of synthetic antioxidants as oil preservatives has been restricted in many countries due to their adverse health effects, thus increasing interest in natural alternatives. Antioxidants from plant extracts have been found to be promising in preserving other oil-focused products, such as biodiesel. The utilization of olive leaves by converting them into natural antioxidants can significantly reduce the adverse effects of olive oil production on the environment. Khounani et al. (2021) used olive pomace to produce olive pomace oil, which is then converted into biodiesel. This work proved that environmental impacts per ton of olive oil produced could also be reduced by using olive leaf methanolic extract (OLME) as a natural antioxidant to extend the shelf life of the produced olive pomace oil biodiesel fuel [25].

The potential use of lignocellulosic bio-oil as an antioxidant to protect biodiesel from autoxidation is also reported by Garcia et al. (2017) with promising results [26]. In addition to edible oils, natural phenolic compounds can also be produced from inedible plants. Since lignin is the only renewable polyphenolic polymer, it has a potential substitute for petroleum-based phenolics [26].

The bio-oil produced by the pyrolysis of lignocellulosic biomass has a different chemical profile. In addition to a wide known range of applications related to the synthesis of pharmaceutical and food additives and the production of adhesives and resins, the addition of bio-oil to biodiesel has resulted in an increase in the oxidation induction temperature. The inclusion of less than 4 wt.% concentration of bio-oil compounds by Garcia et al. (2017) led to a 475% improvement in biodiesel oxidation stability. Thus, the authors proved that acetate esters are the best option for incorporating bio-oil antioxidant compounds into biodiesel [26].

**Figure 2:**
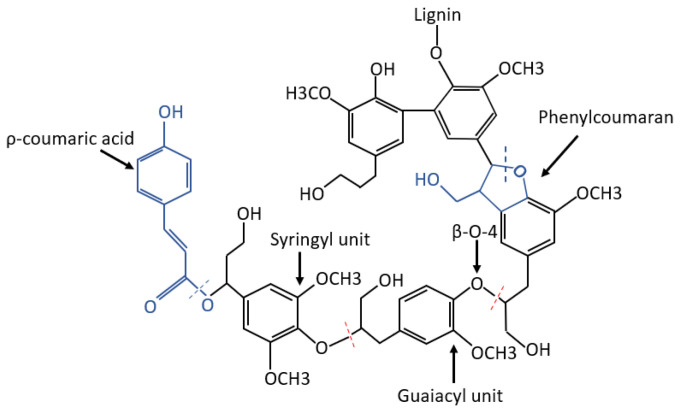
Structural transformation of lignin depolymerization by An et al. (2019) [23].

Lignin is a heterogeneous polymer that attracts great attention because of its antioxidant activity due to its polyphenol structure. In An et al. (2019), the depolymerization of high molecular weight lignin through moderate ethanol/acid catalysis was performed to improve its solubility and antioxidant activity. Depolymerization resulted from the cleavage of ether linkages that decreased the molecular weight in an 80% ethanol-water solution. The model structure of lignin depolymerization is shown in Figure 2. The presence of ethanol is important in suppressing lignin condensation, evidently enhancing the depolymerized products’ contents. The authors proved that lignin is a promising candidate as a commercial antioxidant since the 1,1-diphenyl-2-picrylhydrazyl (DPPH) free radical scavenging capacity of depolymerized product at 200 °C (D-200) was close to that of butylated hydroxy-toluene (BHT). The authors’ work confirmed that moderate depolymerization of lignin by ethanol/acid catalysis effectively enhances solubility and antioxidant activity [23].

## 3. Antistatic Agents

Antistatic agents (or antistats) are added to polymers to concede some electrical conductivity to avoid or minimize the accumulation of electric charges in the final plastic materials. This electric charge accumulation can turn the polymers exposed to static clings, electric discharges and dust adhesion [27,28].

Depending on their application, antistatic agents can be classified as external and internal. External (or topical) antistatics are typically surface-active ionic or nonionic antistatic agents and are usually applied to the polymer surface (e.g., for fibers and film surface treatments), where the hydrophobic part is in contact with the polymer and the hydrophobic counterpart is directed to the air. These antistatic agents also prevent dust from falling on the surface of the polymer, even if they can leave the polymer through regular use, such as by rinsing the material. They are commonly used where a short-term antistatic effect is necessary (plastic packages for electronic parts, for example). Internal antistatic agents are incorporated into the polymer during its manufacturing to develop conductive paths through the material, allowing discharges from the surface to the ground or boosting at the surface to allow electron dissipation in the surface layers. This type of antistatic agent is applied, for example, to the manufacture of polyolefins, PS, PVC and acrylonitrile-butadiene-styrene (ABS) polymers. Cation-active quaternary ammonium, phosphonium or sulfonic salts or anion-active sulfonates and phosphoric acid derivatives are external anti-stats. Fatty acid esters, polyhydric alcohols or fatty amine polyglycol ethers are examples of typical internal antistatic agents. However, some materials can be applied as internal or external antistatic agents such as conductive (doped) polymers (e.g., poly-pyrrole, polyphenylene and polyaniline) [27,28].

Based on their chemical structure, the organic antistatic agents can be classified as nonionic, anionic, cationic and amphoteric (Table 3). Salts, metals, semiconductors, carbon black, carbon nanotubes and other intrinsic conductive or conductive modified particles are common inorganic antistatic agents. They are frequently applied for a bulk modification to add antistatic properties or conductivity to the material [27,28].

The antistatic agents can be incorporated into the polymers using different methods [27]:

Grafting—This is an economical method to promote antistatic properties. The antistatic additive is added in small quantities and will prevail on the polymer surface despite polymer treatments such as irradiation, washing and migration [27]. To avoid charge accumulation on the surface of polyolefins (since their surface is usually hydrophobic), poly-2-hydroxyethyl-methacrylate was grafted to the surface of PP through the vapor phase photochemical grafting method. The hydrophilic layer formed highly improved its antistatic properties [29].

Chemical modification—By modifying the structure and surface functionality of a carbon black (CB) through gasification with carbon dioxide, it was possible to decrease the electric resistivity of PE containing the modified CB [30]. Another work using this method was the one promoted by Chen et al. (2005) [31], who synthesized hydrophilic poly(oxyethylene) (POE) and olyoxy-propylene blocks with chlorotriazine linking groups to prepare flexible films with low surface resistivities suitable for use in dissipation applications.

Surface coating—In this method, only the coated layer receives the antistatic agent, and it is considered the second most economical method. However, several problems can occur, such as the migration of the additive to the bulk of the material, no adhesion between layers and a requirement for supplementary operations [27]. There are several types of antistatic coatings, such as ionomer salts of polystyrene-sulfonic acid or polyacrylic acid and a hydrophilic binder (used in films) [27]; poly-pyrrole (used in the production of yarns and fabrics) [32]; quaternary ammonium salts (applied in the production of conductive coatings) [33]; and inorganic conductive additives, such as titanium oxide (TiO_2_), silicon oxide (SiO_2_) or indium tin oxide (ITO) (for the manufacturing of antistatic and antireflective coatings) [34].

UV and electron beam curing—This technology has gained some industrial interest since it is more energetically efficient, easy to produce and has short curing times [27,35]. To confer antistatic properties, an organic-inorganic acrylic coating was made using a trialkoxy-silyl ammonium salt in the photocurable formulation [36]. Roessler and Schotteberger [37] studied the antistatic effects of ten imidazolium-based ionic liquids (ILs), using this technique to produce antistatic coatings for wood floorings, where the coating with 3 wt.% 1-allyl-3-methylimidazolium chloride demonstrated a significant anti-dust effect.

Plasma treatment—The textile industry has recently become more interested in plasma treatment technology, since it appears to be a promising, environmentally friendly, and cost-effective replacement for traditional wet-chemical processing methods. Plasma surface treatment is a reasonably straightforward, clean, solvent-free and time-saving procedure. Additionally, plasma treatments provide the chance to attain standard textile finishes without altering the essential textile qualities [38]. Due to their inertness and lack of adhesion, polyolefins were the main application for this treatment. Plasma was used to modify polyolefin surfaces by oxidization and becoming hydrophilic. Dehydrogenation, radical generation and surface roughening are additional effects of plasma treatment [27,39].

Physical Vapor Deposition (PVD)—Thin film coatings on fabrics and fibers can be produced via PVD [27]. Several titanium-based coatings were produced by PVD using the arc deposition mode in polyamide fabric. The developed coatings appeared promising for increasing the fabric’s antistatic properties [40].

Mixing/dispersion—This method is one of the processes that generate static electricity. During simple mechanical processes such as molding or extrusion, a charge is produced on the surface of the polymer as it separates from the surface of the metal in contact. Static electricity can also be generated when flowing liquids encounter solid particles. This can also happen when different sizes of solid particles are mixed into liquid in insulated containers [27]. The dispersion of carbon nanotubes—CNT (single-walled carbon nanotubes—SWCNT or multi-walled carbon nanotubes—MWCNT) and carbon nanofibers (CNF) in a polymer matrix are examples of this technique [41,42,43].

Crystallization—in matrix–transparent and electrically conductive materials can be obtained through the crystallization of conducting charge-transfer complexes (CT-complexes) in a polymer matrix [44].

Nucleation of inorganic nanoparticles—Polyaniline nanofibers (with diameters between 30 to 120 nm) were used as a template for the nucleation of gold nanoparticles to improve the electrical conductivity of the nanofibers [27].

Antistatic agents have been widely employed in different areas of industry: adhesives and sealants, automotive, agriculture, aerospace, construction, fabrics, fibers and textiles, composites, cosmetics, pharmaceutical and medical products, electrical equipment, foams, footwear, membranes, paints and coatings, paper, tires and packaging [27]. CB is the most commonly used antistatic agent, giving the finished polymeric composite its black color and conductivity. These polymeric composites have a wide range of uses, including electromagnetic interference shielding and electrostatic discharge [27,45,46]. CNT has also been widely studied to improve not only the antistatic characteristics of polymers (e.g., PS, polyethylene-butene matrix, poly(methyl methacrylate), epoxy polymers and acrylonitrile-styrene–butadiene), but also their mechanical and thermal properties [45,47,48]. Dodecyl-benzenesulfonate doped polyaniline/organoclay nanocomposites and propylene–ethylidene–norbornene rubber were studied for their application as antistatic agents and electromagnetic shielding [49]. Zinc oxide (ZnO) is usually added to PS resin to improve the antistatic properties of the materials and lower the surface resistivity [50].

ILs have been widely studied due to their chemical and thermal stability, low flammability and negligible vapor pressure. Due to this last characteristic, ILs, for many years, was considered to be non-toxic, although it has been proved that this is not true for some of them, especially for ILs with cations with long alkyl-side chains [51]. Besides that, ILs have been widely studied in different areas including in the plastics field (e.g., as photo- and thermo-responsive materials, antistatic agents or plasticizers) [52]. They have gained popularity as antistatic agents in recent years. When applied to the polymer’s surface, these materials decrease their potential to conduct static electricity [53]. The IL, 1-butyl-3-methylimidazolium bis(trifluoro-methane-sulfonyl)-imide ([C_4_mim][Tf_2_N]) was added to polyether-based polyurethane films to enhance their antistatic properties long-term [54]. Tsurumaki et al. studied the antistatic effects of several ILs in different polyether-based polyurethanes and polymethacrylates [55,56,57]. To avoid the tendency to electrostatic charge in wood floorings, various methylimidazolium-based ILs were studied as potential antistatic agents. The 1-allyl-3-methyllimidazolium chloride has the maximum activity and the lowest leaching rate in floor coating [37].

### Bio-Based Antistatic Agents

In recent years, there has been a growing awareness of the materials used in industry. There have been few or no studies on the toxicological impacts of most antistatic agents used in the environment or in human health, and the few existing studies show that some of these antistatic agents can cause cancer and/or severe respiratory inflammation such as asthma, rhinitis, sinusitis or nasal septal perforations [27].

Bio-based materials have aroused the scientific community’s interest due to their biodegradability, renewability and non-toxicity. Natural polysaccharides derived from different sources (agricultural and forestry wastes, for example) are an important renewable resource. They are particularly versatile due to their chemical composition and structure diversity. They find their application as substitutes for petroleum-based polymers in various industries, including tissue and textile engineering, food, food packaging and bioplastic production [9].

Grapes are a significant fruit that are consumed in large quantities. Winemaking is the most typical application for grapes that every year generates large quantities of solid waste, representing approximately 25–30% of grapes. This biomass mainly consists of grape skins and seeds, which are high in phenolic compounds, such as phenolic acids, catechin, anthocyanins and flavonoids (for example, proantho-cyanidins). Grape seed proantho-cyanidins (GSPs) are polymerized flavonoid compounds known as condensed tannins, with flavan-3-ols as their monomeric components [58]. GSPs have been studied for dyeing, flame-retardant qualities, and antibacterial treatment of silk [58] and as a bio-based coloristic, anti-pilling, antistatic additive for cashmere [59]. In this last application, the authors presented a one-pot, high-efficiency, low-temperature ultrasound-assisted approach for coloristic, anti-pilling, antistatic, bioactive and reinforced cashmere utilising GSPs. When compared to the usual water bath treatment, the adsorption efficiency increased by 56% at 60 °C and 101% at 80 °C using ultrasonic technology. The authors also observed that the high effectiveness of GSPs as antistatic agents was similar to that of quaternary ammonium antistatic agents on wool. The modest color fading (<10%), pilling rating drop (within one level), and static half-period (still less than 2 s) after 20 washing cycles demonstrated the high durability of GSPs on cashmere fabric [59].

Chitin, like cellulose, is one of the most natural and environmentally beneficial materials on the planet and consists of *β*-(1→4)-linked-2-acetamido-2-deoxy-b-d-glucose (*N*-acetylglucosamine). Chitosan is a modified hydrophilic carbohydrate polymer derived from chitin and found naturally in the exoskeletons of marine crustaceans, shrimp and crabs. It may also be recovered via alkaline *N*-deacetylation of fungal cell wall chitin and chitins from simple arthropods. Because of its antibacterial and fungal characteristics, it is commonly used to treat skin infections and as a drug carrier in modern treatments. Chitosan’s antibacterial activity, as well as its combination with other polymers, have been investigated for a variety of Gram-positive and Gram-negative bacterial infections. Biodegradability, biocompatibility, non-toxicity and physicochemical properties are the most important characteristics of chitin and chitosan [60].

Zhang et al. (2019) [61] used silk fibroin/chitosan microspheres (SFCM) and low-temperature nitrogen plasma (LTNP) to enhance the antibacterial and antistatic capabilities of polyethylene terephthalate (PET) fabrics. First, silk fibroin and chitosan were combined to produce microspheres. Then, LTNP was used for PET warp-knitting fabrics. After that, the LTNP-treated fabrics were absorbed by SFCM at room temperature. The results showed that the antistatic and hygroscopic properties of the LTNP-SFCM-treated PET fabrics were considerably enhanced. The LTNP-SFCM-treated PET fabrics also showed outstanding antibacterial properties against *Staphylococcus aureus* and *Escherichia coli*.

Cellulose is nature’s most abundant and renewable organic biopolymer resource. It is composed of linear chain *β*-1,4-O-glucoside linked with D-glucose forming long linear hydrogen bonds connected by intermolecular Van der Waals forces. Microorganisms may decompose in a short period without causing harm to the environment. As a result, it is biodegradable, biocompatible, and hemo-compatible. Cellulose can be defragmented to produce several derivatives such as cellulose nanofibers, cellulose diacetate, cellulose acetate phthalate (CAP), cyanoethyl cellulose (CyEC) and methyl cellulose [62,63,64,65,66]. CAP is one of the most promising polymers for improving the filtration properties of membranes, providing an appealing route for producing sustainable materials capable of meeting the growing demand in this field. The combination of quaternized poly-sulfone (PSFQ) with CAP led to the development of a novel method for producing membrane materials with long-term stable hydrophilicity, better workability, porosity, and good biocompatibility.

Additionally, the quaternization effect has significantly enhanced the electrical performances required by target applications, such as ionic-exchange membranes, in terms of ionic conductivity, electron interactions and dielectric properties [64]. To enhance the dielectric properties of CyEC, the residual hydroxyl groups were esterified and then derivatized with multiple substituents with molecular and local segmented orientation induced, which was performed by a uniaxial heat-stretching process. The results have shown that the esterification with longer chain fatty acids originated from less-order materials, although thermally moldable films were successfully obtained. This novel approach for creating an electrically functionalized cellulosic material, with chemical modification followed by thermal processing, can be an effective and viable alternative [65]. Methylcellulose has excellent features, such as water solubility, non-toxicity, availability, low cost, biocompatibility and biodegradability. Furthermore, the methylcellulose inter-molecular structure generates a 3D network of formed hydrogen bonds with niacin or carboxylic acid additions, which increases the dipole moment. Hasanin and Labeeb (2021) [66] proposed a study that presented nicotinic acid as a composite with methyl cellulose to improve the dielectric characteristics of surfaces with persistent antistatic charge. The preparation procedure, suggested by the authors, is basically “green,” requiring only highly filtered water. The composite is a powder that can be easily collided and sprayed on surfaces to make them antistatic charge surfaces.

## 4. Plasticizers

Plasticizers are the most widely used additives in the plastics industry. They are primarily liquid and typically non-volatile organic compounds. When added to a plastic or an elastomer, they can improve the polymer’s flexibility, extensibility and processability. These enhancements result from the plasticizer’s dissolution in the polymer, which may also lower the end product’s elastic modulus or glass transition temperature.

The increase of a material’s plasticity is often required in the production of plastic commodities and/or packaging, epoxy resins (coating cans for food and beverage), water pipe lining, thermal printing paper, implanted medical devices, CDs and DVDs, mobile phones, eyeglass lenses, drinking bottles, food packaging, dental sealants, among many others [67,68,69].

Some of the most widely used plasticizers are elastomers, particularly non-polar elastomers; these are petroleum oils, which include aromatic, naphthenic and paraffinic petroleum products. Natural rubber, styrene-butadiene rubber (SBR), ethylene propylene rubber (EPR), ethylene propylene diene monomer rubber (EPDM) and neoprene [70] are among the materials in which they can be employed. Polar elastomers, such as PVC, use other types of plasticizers (usually esters), such as phthalates, adipates, glutamates, sebacates, phosphates, polymerics, tri-mellitates, and epoxy compounds [70,71]. Other than having a plasticizing effect, some additives (such as chloro-paraffins and ionic liquids) are multifunctional and have unique properties, such as an antistatic or flame retardancy effect [52,72].

Depending on their characteristics, plasticizers can be divided into two groups: primary and secondary. Since the primary plasticizers can quickly gel the polymer and have solubilities close to those for the polymer, they rarely leach out of the plasticized material. The secondary plasticizers group’s compatibility with polymers is generally quite limited. Therefore, primary plasticizers can typically be used as the only component in the plasticizer formulation, while secondary plasticizers are never applied alone. However, the division of plasticizers is typically based on their chemical structures, phthalates and non-phthalates, and are described in more detail below [12,13].

### 4.1. Phthalates

**Figure 3:**
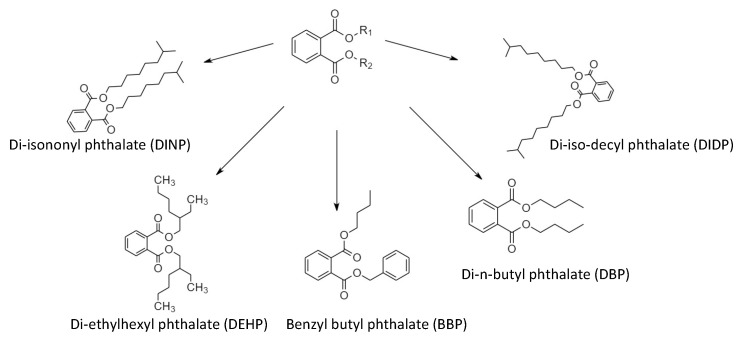
Example of typical phthalates plasticizers, adapted from Marturano et al. (2017) [12].

Phthalate plasticizers, mainly petroleum-based products, are colorless liquid phthalate esters, as shown in Figure 3, that are soluble in most organic solvents, but are unfortunately also soluble in some body fluids [12]. This group of plasticizers can be found in various products, such as clear food wraps, detergents, soaps and pesticides [67].

One of the most commonly used plasticizers was diethyl-hexyl phthalate (DEHP). However, substantial questions have been raised about its harmful effects. Furthermore, according to Marturano et al. (2017) [12], DEHP is not chemically bound to PVC, so it is easily susceptible to leaching, migration or evaporation, which can expose human beings directly through use or contact, and indirectly through leaching into other products, or through environmental contamination [12,13,73]. Since most phthalate plasticizers are hazardous and can disrupt the human reproductive system, it is now recognized that phthalate exposure poses a risk to human health [73].

Epoxidized vegetable oils are one of the phthalate ester substitutes that are actively encouraged, since they are a resource that is sustainable and environmentally friendly. However, the epoxidation process is quite expensive and often employs a non-reusable catalyst, making it a less-than-optimal method [74]. As a result, the need for an alternative to phthalate derivatives arose.

### 4.2. Non-Phthalate Plasticizers

Phthalates, considered the most dangerous plasticizers, have been gradually phased out in the EU due to tighter regulations [75]. As a result, the need for another set of plasticizers was considered, and non-phthalate plasticizers began to replace the earlier ones. In this group are the phosphoric esters, adipates or sebacates, tri-mellitates and benzoate esters, which are currently considered to be a less toxic alternative to phthalates. However, the most extensively researched, most prevalent, and possibly best-known non-phthalate plasticizers are also emerging pollutants. According to Barceló and Petrovis (2008) [76], bisphenol A (BPA) and its derivate bisphenol A diglycidyl ether (BADGE) are substances that can leach into the environment and are present in both plastic baby bottles and metal food and drink cans [67]. BPA has also been shown to be harmful to animal development, particularly in males [77,78,79,80], as it alters the development of the prostate, reduces sperm counts and disrupts the body’s hormonal balance. As a result, even if many of these alternatives have a tremendous potential application, prolonged exposure could have harmful impacts on one’s health [12,75,81]. Additionally, because all the plasticizers mentioned are based on petrochemicals, research has focused on finding bio-based alternatives with comparable mechanical qualities [12,82].

### 4.3. Bio-Based Plasticizers

Environmentally friendly source materials for production have proliferated in recent years [83,84] and different natural sources, such as starch, can be used individually or combined, glycerol, polyols (such sorbitol and xylitol) and vegetable oils being the most commonly used plasticizers, along with water [85].

Some commercially available plasticizer formulations, such as Syncroflex TM by Croda, based on esters of di-fatty acids, have proven to be patentable [86]. For example, Lim et al. (2015) [87] reported the use of palm oil-based alkyd in PVC as a co-plasticizer to dioctyl phthalate (DOP) and di-isononyl phthalate (DINP), resulting in an enhancement of the mechanical and thermal properties of the final formulation. Using epoxidized sunflower oil as a secondary plasticizer for PVC in conjunction with DEHP was also successful, according to Bouchareb and Benaniba (2008) [88]. Oleic acid was used to create the cold-resistant bio-based plasticizer, and the plasticized PVC showed impressive migration and cold resistance [89].

Besides PVC applications, natural-based products have received extensive reviews as plasticizers for biopolymer films of various natures. These include polysaccharides (starch, cellulose, pectin and chitosan), microbial polyhydroxy-alcanoates (PHAs), polyhydroxy-butyrates (PHBs), and polylactic acid (PLA). Additionally, employing natural plasticizers ensures that the finished product is environmentally sustainable. Due to their capacity to function as phyto-colloids and as emulsifying agents, for example, alginates, they are widely used in the food sector and in the pharmaceutical and medical fields. Furthermore, they are frequently used in the form of polymer composites or blends for food packaging applications [90,91]. Given this polymer, it is crucial to adjust and perfect the physicochemical characteristics for the intended uses, being common knowledge that the application of sodium alginate films is severely constrained by their brittleness and fragility [89,91]. To enhance the capabilities of the alginate and broaden its possible applications, researchers have concentrated on the usage of plasticizers over the past ten years [92,93,94].

The use of waste biomass from the food industry and/or agricultural byproducts as plasticizers for biopolymers is auspicious. More specifically, Di Donato et al. (2020) [9] reported that polysaccharides extracted by green processes from waste biomass of the lemon and fennel food industry could be handled as plasticizers for sodium alginate films, thus offering a circular flow of secondary-raw materials. This suggests that using a natural plasticizer such as sodium alginate could show an excellent example of an eco-sustainable, cost-effective circular flow of renewable materials, in contrast to synthetic polymers of original origin [9].

Using natural plasticizers in bioplastic production is a good alternative for the future. It can become a suitable replacement for petroleum-based plastics and an acceptable method to moderate the plastic pollution problem after comparing bio-plasticizers with conventional plastics production [95].

## 5. Conclusions

The awareness of the need to control and decrease climate change and greenhouse gas emissions has become a priority for academic research, governments and industry. Renewable source materials have been studied and used as substitutes for petroleum-derived products and materials.

In the polymeric materials field, the search for greener polymers has not only focused on developing new materials but also on using biomass to produce additives (such as antioxidants, antistatic agents and plasticizers) in order to produce greener, biodegradable and less toxic materials. In this review, studies were presented where agro-industrial wastes, such as grape residues (seeds, stems and peels), olive tree and green tea leaves, citrus peels, red-skinned apples, cranberries, carobs, brown-skinned potatoes and red-skinned onions) were used to produce antioxidants to be incorporated into the polymerization process. The lignin antioxidant potential was also presented, as well as the use of bio-oils (produced from the pyrolysis of lignocellulosic biomass) to protect biodiesel from autoxidation. Grape residues (mainly skin and seeds), chitin and chitosan and cellulose derivatives were some renewable materials with good potential to be used as antistatic agents in polymeric materials. The potential of agro-industrial wastes, polysaccharides (such as cellulose, chitosan, pectin, and starch), polyols, glycerol and vegetable oils as bio-sourced materials for the production of plasticizers, was also demonstrated.

This review shows the great potential of biomass residues and bio-source materials in producing environmentally friendly polymeric materials, although more studies are needed, mainly regarding the biodegradability and toxicity of the final products.

## Figures and Tables

**Table 1:** Classification of some commercial AOs and their chemical composition by Ambrogi et al. (2017) [13].

Classification	Chemical Composition	Supplier	Implementations
Primary AOs	Phenols	ANOX 29 (Addivant), IRGANOX 1010 (BASF), EVERNOX 10 (Everspring)	Polyvinyl chloride (PVC), Polyamide (PA), PP, PE, cellulosic polymers
		ADK STAB A040 (AdekaCorp), SONGNOX 1077 LQ (Songwon)	Cellulosic polymers
	Amines	AMINOX (Addivant), DUSANTOX 86 (Dulso), ANTIOXIDANT DQ (Akrochem)	Natural rubbers
		Sipax DLTDP, BNX 2000 (Mayzo)	PA, PE, PP
Secondary AOs	Phosphites	WESTON 705 (Addivant), ADK STAB 1500 (Adeka Palmarole)	Cellulosic polymers
		EVERFOS 168 (Everspring Chemical), ADK STAB PEP-36 (Adeka Palmarole), ALKANOX 240 (Addivant)	PVC, Polystyrene (PS), PA, PP, PE, cellulosic polymers
	Thioester	Octolite 529 (Tiarco Chemical)	Synthetic rubbers
		Songnox DSTDP (Songwon), Irganox PS800 (BASF)	PA, PE, PP, PVC, PC

**Table 2:** Use of natural antioxidant compounds.

Plant Source	Phenolic Components	Ref.
Grape seeds	Gallic acid, proantho-cyanidins and flavanols	[21]
Grape stems	Flavanols, proantho-cyanidins, flavonols and hydroxycinnamates	[21]
White grape peels	Flavanols, proantho-cyanidins and hydroxycinnamates	[21]
Red grape peels	Anthocyanins, flavanols, proantho-cyanidins, flavonols and hydroxycinnamates	[21]
Apples (red-skinned)	Flavanols, proantho-cyanidins, flavonols and hydroxycinnamates	[21]
Potato (brown-skinned)	Chlorogenic acid	[21]
Onion (red-skinned)	Flavonols, anthocyanins	[21]
Olive tree leaves	Oleuropein, flavones, flavonols, flavonol derivatives	[21]
Carob	Gallic acid, gallo-tannins, proantho-cyanidins, flavanols, flavonols	[21]
Citrus peels	Flavonoids, carotenoids, pectin	[20]
Green tea	Flavanols	[20]
Cranberries	Flavonoids, phenolics and fiber	[20]

**Table 3:** Examples of common organic antistatic agents [27,28].

Classification	Examples
NONIONIC	Fatty acid esters Fatty amine polyglycol ethers Fatty acid diethanolamides Fatty alcohol polyglycol ethers
ANIONIC	Alkyl sulfonates Phosphoric acid alkyl esters
CATIONIC	Quaternary ammonium compounds
AMPHOTERIC	Alkyl betaines
